# An analysis of different reconstruction techniques for the radius: a finite element analysis

**DOI:** 10.1007/s10237-026-02116-z

**Published:** 2026-09-28

**Authors:** U. Konopada, V. Egger, L. Dankl, M. Nogler, R. Arora, E. Zanetti, C. Bignardi, D. Putzer

**Affiliations:** 1 Department of Mechanical and Aerospace Engineering, Politecnico Di Torino, Corso Duca Degli Abruzzi, 24, 10129 Turin, Italy; 2 Department of Orthopaedic Surgery and Traumatology, Inselspital University of Bern, Freiburgstrasse 20, 3010 Bern, Switzerland; 3 Department of Orthopaedics and Traumatology, Medical University of Innsbruck, Anichstraße 35, 6020 Innsbruck, Austria; 4 Department of Orthopaedics and Traumatology, Experimental Orthopedics, Medical University of Innsbruck, Anichstrasse 35, 6020 Innsbruck, Austria; 5 Department of Engineering, Università Degli Studi Di Perugia, 06125 Perugia, Italy

**Keywords:** Polymethylmethacrylate (PMMA), Bone reconstruction, Cementoplasty, K-wire reinforcement, Plate fixation, Musculoskeletal modeling

## Abstract

Pathological fractures due to metastatic bone disease frequently require palliative stabilization. While polymethylmethacrylate (PMMA) provides immediate mechanical stabilization, its limited bending and multiaxial properties raise concerns. Reinforcement strategies such as plate fixation, intramedullary nails, and metallic augmentation have been proposed, yet their mechanical behavior under physiological loading remains insufficiently characterized. This study aims to evaluate the mechanical performance of three reconstruction strategies for a proximal radial defect: PMMA alone, PMMA combined with titanium plate-and-screw construct, and PMMA reinforced with a steel K-wire. A patient-specific workflow integrated musculoskeletal multibody model and finite element analysis. Muscle forces and elbow joint reaction forces were calculated for a static 90° shoulder flexion under two loading conditions: no external load and 2 kg mass applied at the center of mass of the hand. These forces were applied in finite element analysis in reconstructed forearm, and peak von Mises stress and displacements were evaluated. In loading scenario, elbow joint reaction force reached 314.4 N, and maximum muscle force was 120.8 N. In the PMMA-only construct, peak stress increased from 4.0 MPa (no load) to 12.0 MPa (with load). Plate fixation increased peak stress to 14.9 MPa in the loading case. K-wire reinforcement resulted in lowest PMMA peak stress (23% less with respect to PMMA-only and 38% less compared to titanium plate) but produced more pronounced displacement (29%) compared to plate fixation strategy. These findings suggest complementary mechanical benefits: K-wire reinforcement most effectively reduced peak cement stress, whereas plate fixation provided superior global stiffness.

**Supplementary Information:**

The online version contains supplementary material available at https://doi.org/10.1007/s10237-026-02116-z.

## Introduction

Pathologic fractures (complete or impending) appear in bones weakened by an underlying process caused by a disease and result from insignificant trauma. They occur most commonly in patients with bone metastases from cancer, which is estimated to be approximately 500 times more frequent than primary bone tumors (Biermann et al. 2010). Nevertheless, primary malignant bone tumors, benign bone lesions, and tumor-like conditions must also be considered in the differential diagnosis. Pathological fractures are associated with significant morbidity and reduced overall survival (Saad et al. 2007). The skeleton represents the third most common site of metastatic disease (following lungs and liver), with metastases most frequently involving the axial skeleton (spine, pelvis, proximal long bones) due to high concentration of hematopoietic red bone marrow (Coleman 2001). Metastatic bone involvement may arise from both hematologic malignancies and solid tumors. Multiple myeloma (MM) is the most common cause of bone metastasis (incidence of 70–95% (Coleman 2001)), with reported risk of pathological fracture ranging from 45% (Cowan et al. 2022). Frequently, pathologic fractures are the presenting symptom that leads to the diagnosis of MM. Solid tumors that are frequently metastasizing to bone include breast cancer (~ 35%) and prostate and lung cancer (~ 18% each) (Saad et al. 2007).

Surgical fixation of complete or impending pathological fractures is primarily a palliative intervention. Operative management is generally indicated to preserve quality of life, given high rates of non-union associated with conservative treatment due to impaired bone healing. The primary goals in metastatic bone disease are pain relief, maintenance of function, and the provision of durable constructs that exceed the expected survival and allow for intermediate use. Achieving these objectives is challenging in the setting of compromised bone biology, insufficient bone stock, and tumor-related segmental defects. Depending on fracture location and patient-specific factors, treatment options include kyphoplasty, arthroplasty, or internal fixation by intramedullary nail or plate osteosynthesis (Damron et al. 2025). Augmentation or defect filling with polymethylmethacrylate (PMMA) bone cement (BC) is frequently required.

PMMA is widely used in orthopedic surgery due to its immediate mechanical stability and high compression strength, pain reduction, ease of handling, and cost effectiveness (Bondi et al. 2025). PMMA can also be loaded with antibiotics: Low doses (< 2 g per cement batch) are typically used for prophylaxis, whereas higher doses (> 2 g per batch) are applied for therapeutic purposes. Studies have demonstrated continuous local antibiotic release for up to approximately six weeks (Coraça-Huber et al. 2025). Antibiotic release as well as mechanical properties differ according to the composition and mixing methods used (Egger et al. 2024). However, some disadvantages have been found, such as cytotoxicity, high polymerization temperature, compromised resistance to complex stress, and weak interface between bone and PMMA (Gong et al. 2022, Mounika et al. 2023).

Intramedullary nails represent the preferred treatment option for lesions in the femoral shaft. In contrast, humeral involvement may be addressed with plate-and-screw fixation as an alternative to nailing. No standards exist for the management of uncommon acral fractures occurring on the forearm or lower leg (Auran et al. 2022, Biermann et al. 2010). Percutaneous cementoplasty is a less commonly employed technique and is typically reserved for patients who are poor surgical candidates; however, in selected cases involving the pelvis, it may serve as an alternative to extensive reconstructive procedures such as total hip arthroplasty (Garnon et al. 2019).

While PMMA provides excellent resistance to compressive forces, it offers limited strength against bending and torsional loads (Kuehn 2013). Thus, cementoplasty alone is less effective on bones that are subjected to multiaxial stress. Different reinforcement techniques have been proposed to improve mechanical properties of bone cement (BC), either by altering the chemical composition or by applying macro-metal reinforcement strategies in clinical practice (Lewis 2008). For humeral metastases, Kim et al. reported minimally invasive treatment using flexible intramedullary nails combined with PMMA, aiming to stabilize the humerus in advanced cancer patients (J. H. Kim et al. 2011). Related work extended this strategy to the lower extremity, showing feasibility of flexible nails and BC (Y. Kim et al. 2014). For femoral neck fractures in high-risk cancer patients, Lin et al. described a technique involving a cement infusible hollow-perforated screw, integrating PMMA with internal fixation principles (Lin et al. 2015). Some recent designs include multi-hole injection nails, providing controlled distribution along the lesion (Park et al. 2019). The proximal femur has received particular attention because cementoplasty alone may be insufficient due to high loads and bending moments. Tian et al. directly compared percutaneous cementoplasty with and without interventional internal fixation, reporting superior outcomes when the fixation was added (Tian et al. 2016).

Another major conceptual reinforcement strategy is the “rebar concept,” where metallic reinforcement is added to PMMA to resist bending and shear. Kelekis et al. described percutaneous augmented peripheral osteoplasty as a combined technique for symptomatic malignant lesions, proposing that reinforcement may improve stability against shear forces during weight bearing (Kelekis et al. 2016).

K-wire augmentation has been proposed as a simple reinforcement method. Cazzato et al. evaluated cadaveric long bones with or without Kirschner wire augmentation in a diaphyseal bending model and reported that the implemented augmentation did not improve resistance to bending stresses in that specific setting (Cazzato et al. 2019). However, the sample size was limited. Putzer et al. performed a controlled comparative laboratory analysis of four-point bending test, evaluating PMMA reinforced with K-wires and PMMA reinforced with pretensioned cables. An improvement in flexural strength in both reinforced groups was detected, demonstrating that K-wire could be a viable macro-metal reinforcement method (Putzer et al. 2024).

In parallel with clinical development, computational modeling has been increasingly used to evaluate the mechanical behavior of cement-based reconstructions, enabling mechanical evaluation under controlled loading conditions. Validated finite element (FE) approaches have demonstrated strong agreement with experimental testing for femurs containing metastatic defects (Sas et al. 2022). Freitas et al. simulated a fall on the greater trochanter before and after femoral reinforcement using FE approach, gaining evidence of increased resistance to fracture causing forces after bone reinforcement (Freitas et al. 2018). Other studies performed FE analysis to investigate different dual locking plate configurations with BC augmentation, indicating an increase in construct stiffness in compound osteosynthesis (Wisanuyotin et al. 2023).

Overall, biomechanical evidence regarding simplified reinforcement approaches such as K-wires remains mixed and appears highly dependent on loading model, lesion location, and reinforcement positioning. While FE modeling has been widely applied to femoral fracture prevention and cement augmentation assessment, such validated models are less frequent in upper-limb reconstruction and physiological loading conditions of reinforced strategies. Therefore, the aim of the present study was to investigate the mechanical performance of PMMA-based forearm defect reinforced with a steel K-wire under physiological upper-limb loading. A computational workflow combining musculoskeletal multibody simulation and finite element analysis was used to compare three reconstruction strategies: BC only, BC with titanium plate-and-screw fixation, and BC reinforced with K-wire.

## Materials and methods

### Case description

**Fig. 1:**
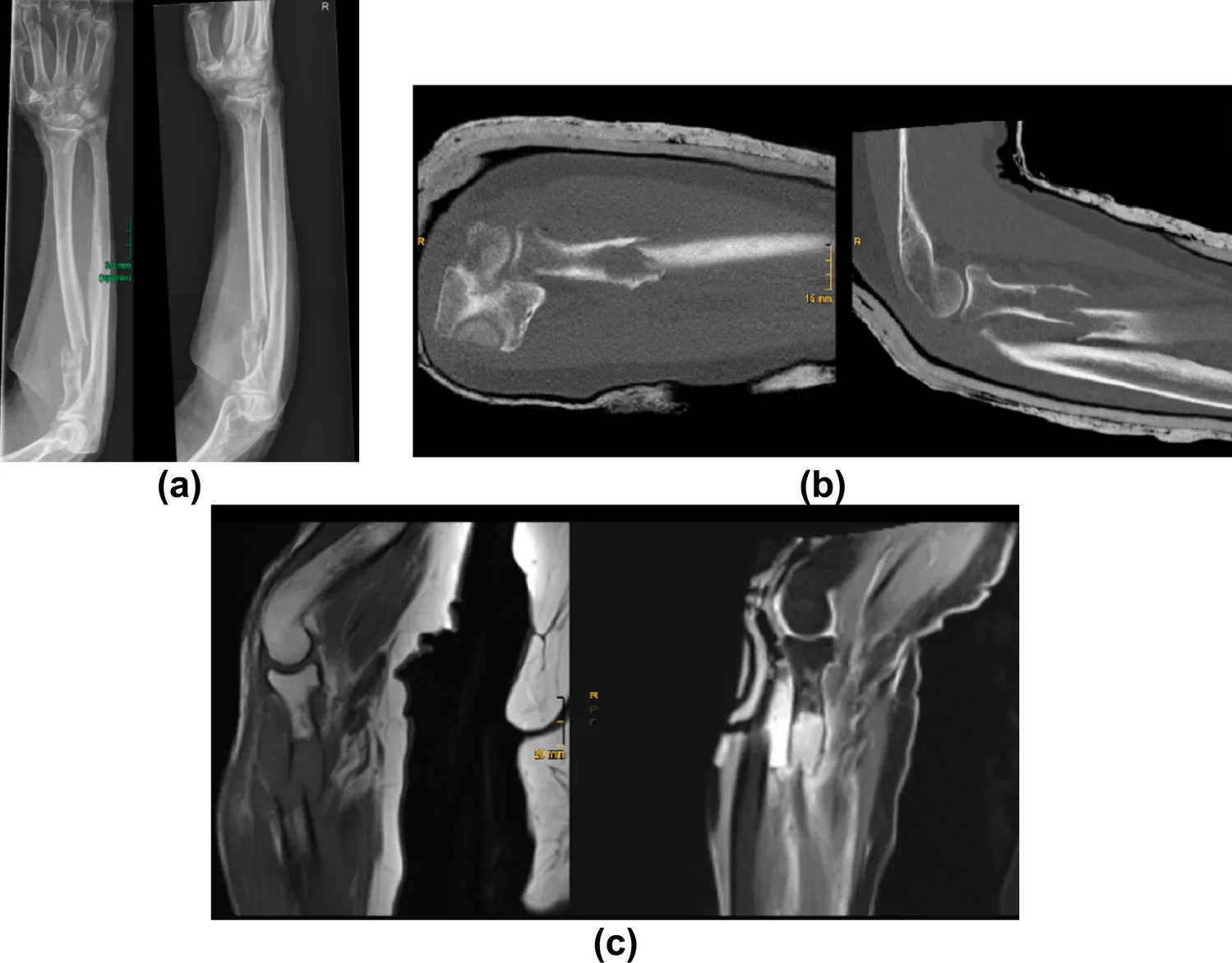
Case description: **a** the a.p. and lateral X-ray after the trauma, where a pathological fracture was suspected; **b** axial (left) and coronary (right) reconstructions of the CT scan, where the osteolysis can be seen; **c** MRI scan with contrast fluid. In the T1 DIXON sequence (right), edema and contrast fluid enhancement can be observed

This study reports the case of an 86-year-old woman, who was referred to a peripheral traumatological emergency room with pain in the elbow after a minor trauma. In the X-ray, a pathological fracture of the proximal radius was suspected. Therefore, a CT scan and an MRI scan were performed which revealed an osteolysis with a longitudinal extent of approximately 30 mm and thinning of the cortical wall (Fig. 1). Further diagnostics with a bone marrow puncture were initiated, which showed an increased kappa/lambda quotient and increased free kappa light chains. In consent with the bone marrow cytology, the diagnosis of a MM was made. Because of the patient’s age and comorbidities, an autologous stem cell transplantation was not possible, and a systemic therapy with daratumumab, lenalidomide, and dexamethasone was started in consent with the latest guidelines on treatment of multiple myeloma (Dimopoulos et al. 2025).

**Fig. 2:**
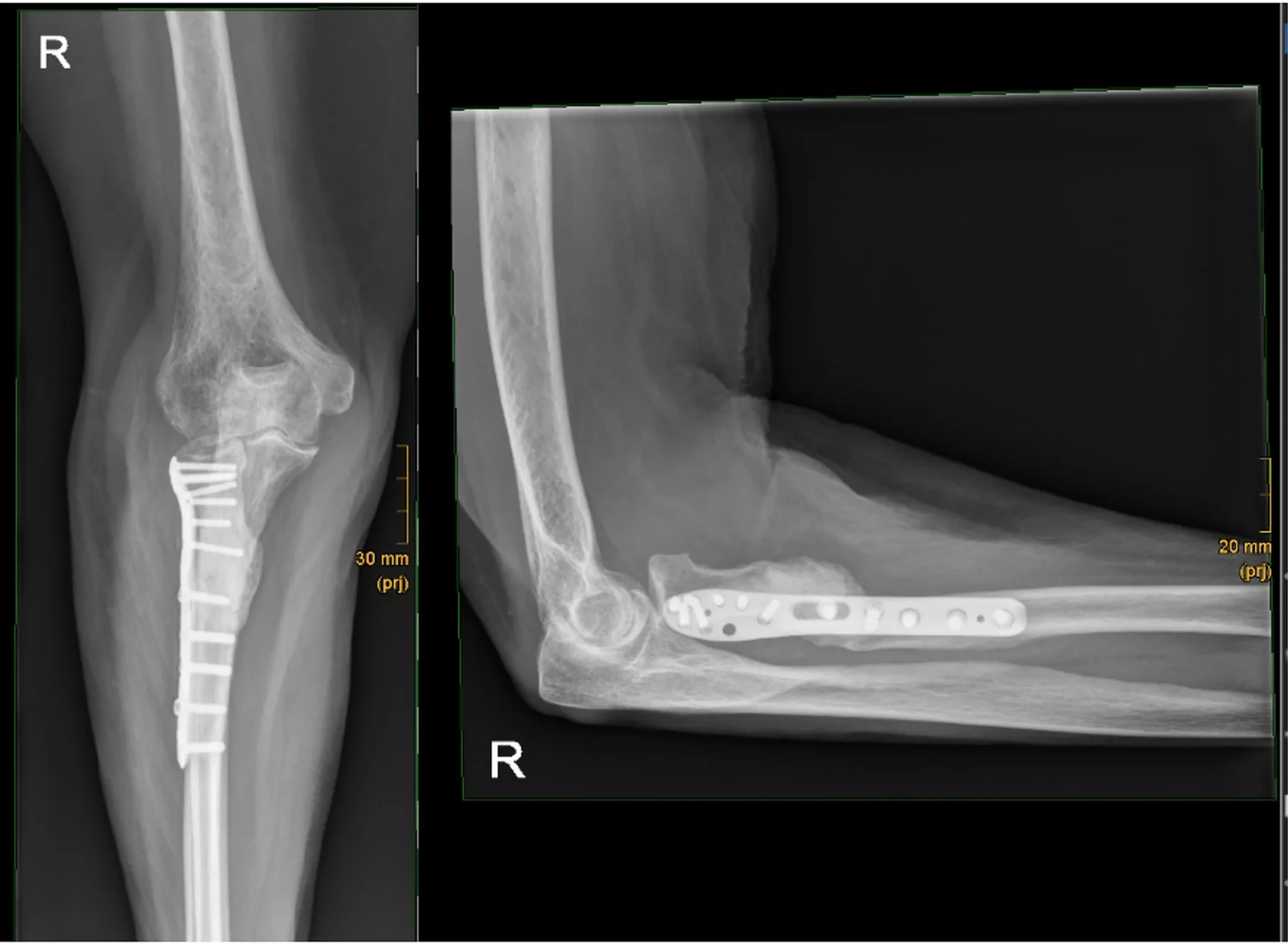
X-ray after compound osteosynthesis with adequate implant positioning and no signs of loosening or implant failure

The patient was referred to our department and discussed in the interdisciplinary musculoskeletal tumor board. In the meantime, the patient had also developed a pathological fracture of the mandible. Therefore, it was decided to perform a compound osteosynthesis with excision of the tumor mass. The operation was carried out accordingly, and the bone defect was filled with bone cement (PALACOS® MV + G, Heraeus Medical GmbH, Germany). Because the defect measured more than 30 mm and the defect started approximately 25 mm distal to the radial head, a conventional proximal radius plate was too short to bridge the gap created by the defect. Therefore, a distal fibula plate (APTUS Ankle 2.8 / 3.5 Distal Fibula Plate, Medartis AG, Switzerland) was used for fracture fixation. Postoperative X-rays showed satisfactory implant position without signs of loosening or implant failure (Fig. 2). An intraoperative tissue biopsy was examined by our department of pathology. The microscopical and immunohistochemical examination showed an infiltration with plasma cells positive for CD138, MUM1, and CD56 as well as negative for CD20 and CD5, matching with a MM. Clinically, the patient showed satisfactory results with normal wound healing and a pain-free full range of motion of the elbow four months after surgery.

### Study overview

The present study employs a computational approach combining multibody musculoskeletal modeling and finite element analysis (FEA) to evaluate the mechanical performance of PMMA-based construct. The clinical case of tumor resection of ulna and radius is presented. The PMMA insert is reinforced with a steel K-wire under physiological conditions. The workflow consisted of three main steps:

1. Estimation of muscle forces and joint reaction forces using a validated musculoskeletal model.
2. Development of finite element models representing different fixation methods and loading conditions.
3. Comparative analysis that focuses on stress distributions within PMMA and different fixation methods.

**Fig. 3:**
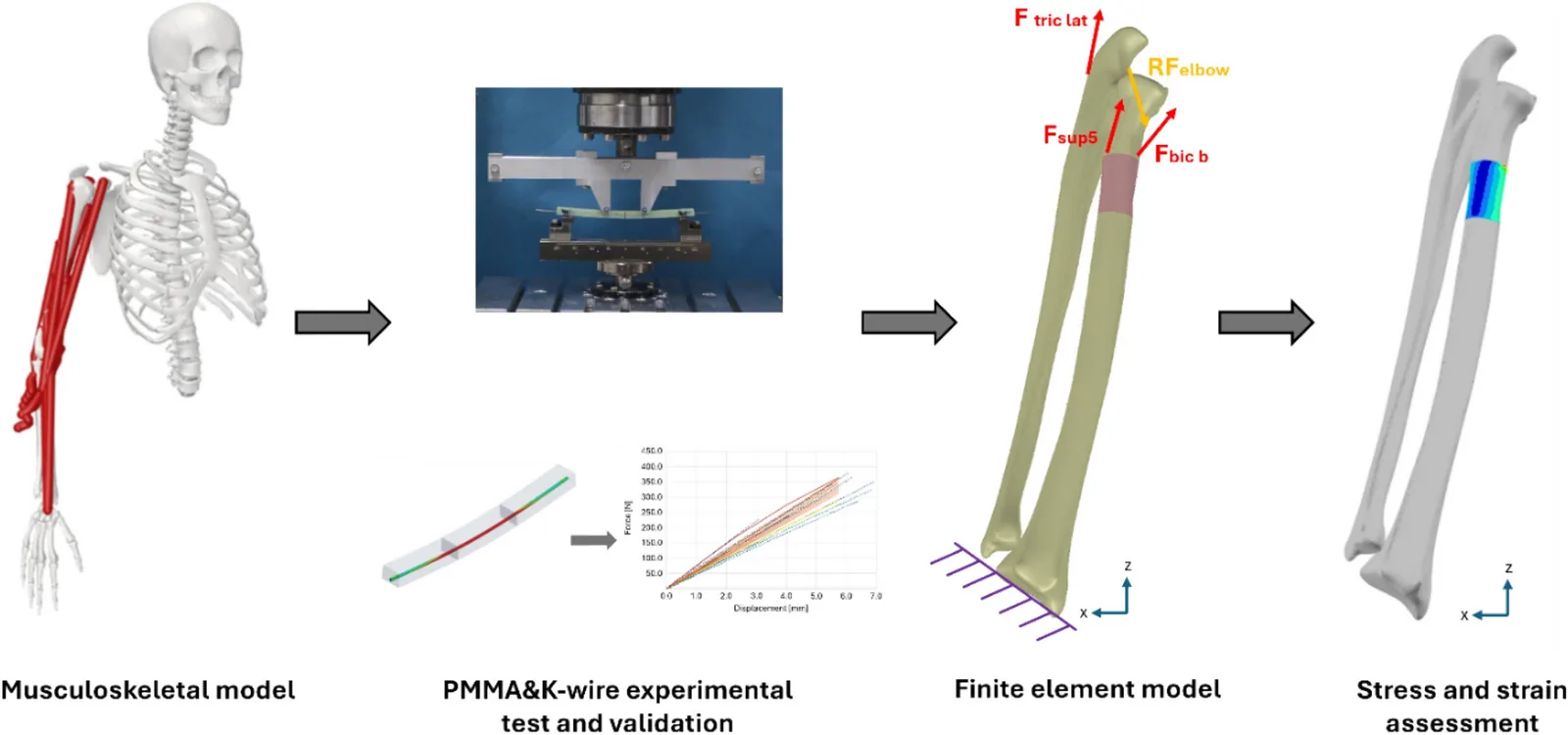
Multibody–FEA workflow

Figure 3 summarizes the multibody–FEA workflow implemented in this study.

### Musculoskeletal modeling and loading conditions

Physiological muscle forces and joint reaction forces were extracted using the Dynamic Arm Simulator (DAS), version 3 (Chadwick et al. 2014), implemented in OpenSim 4.5 (2024, CA, USA) (Delp et al. 2007). The model includes 23 muscles spanning the shoulder, elbow, forearm, and wrist joint and has been previously validated for upper-limb loading scenarios. Muscle actuators are implemented as Hill-type musculotendon units spanning shoulder and elbow. In the DAS model, anatomical muscles are represented by multiple muscle–tendon paths, denoted by numerical numbering. This representation is used to capture physiological muscle multiple lines of action across a joint, allowing a more accurate approximation of force direction, with respect to a single-line muscle representation (Bretler et al. 1999). Consequently, the predicted force output for a given anatomical muscle group is distributed across multiple actuators. For example, biceps brachii is represented by two main actuators bic_b_1 and bic_b_2, each corresponding to a distinct line of action in the DAS model. The triceps brachii medial head is represented by four muscle paths (tric_med_1, tric_med_2, tric_med_3, and tric_med_4). Triceps brachii lateral head is defined by four action lines as well.

A static holding task was simulated with the shoulder flexed to 90° in the sagittal plane. Elbow flexion angle was maintained at 5° and rotation angle at 5°. Forearm and wrist were maintained in neutral position. Two loading conditions were considered: no external load and a negative vertical load of 19.6 N (simulating an object of 2 kg) applied at the center of mass position of the hand. This load was selected to simulate a moderate activity of daily living, corresponding to the handling of common household objects by older adults, while avoiding heavy loading conditions. Similar loading magnitudes have been employed in previous biomechanical studies of upper-limb activities of daily living ((Lee et al. 2015, Anglin and Wyss 2000, Lovern et al. 2010) and are consistent with the light resistance typically introduced during postoperative shoulder rehabilitation and progressive strengthening protocols (American Society of Shoulder and Elbow Therapists (ASSET) consensus recommendations and the Massachusetts General Brigham rehabilitation protocol). No scaling and no morphological modifications of the DAS model were applied. The geometries were registered within the same coordinate system to ensure consistent anatomical orientation, inclination, and reference axes. This alignment enabled accurate transfer of the multibody muscle forces and joint reaction loads to the corresponding anatomical regions in the FE models used to compare alternative fixation strategies.

**Fig. 4:**
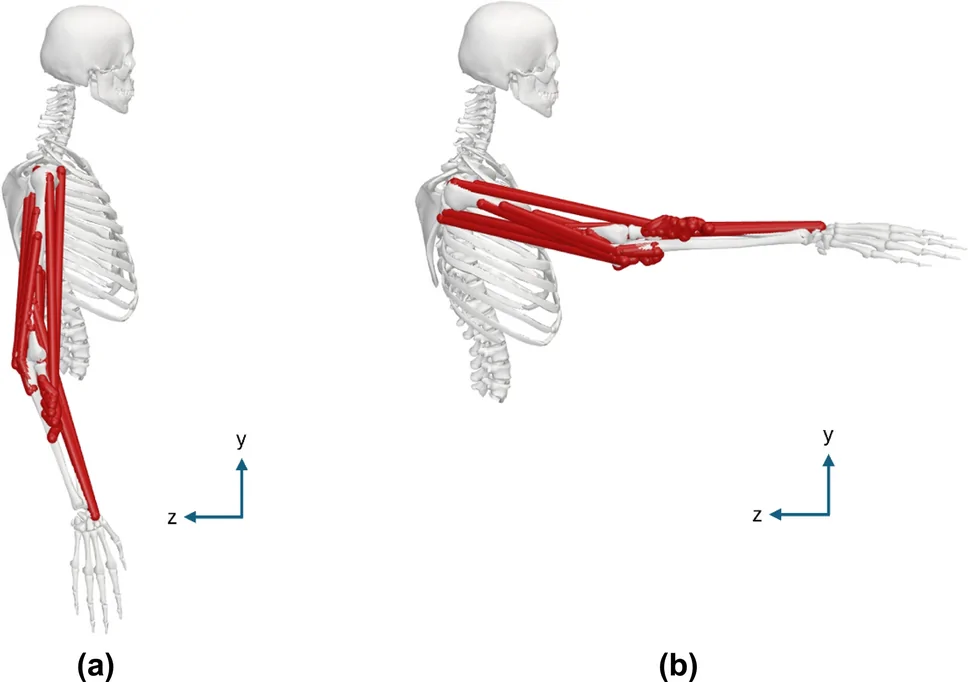
DAS version 3 musculoskeletal model with biceps brachii (long and short heads), brachialis, brachioradialis, biceps brachii (long, lateral, medial heads), anconeus, pronator teres, and supinator muscles: **a** initial position; **b** final position after 90° shoulder flexion

Static optimization was employed to compute individual contribution of upper body muscle forces. The minimum muscle activation threshold was set to 5 N, as these forces are typically negligible compared with primary contributors of the required motion (Avci and Röhrle 2024). Subsequently, joint reaction analysis was performed to obtain wrist and elbow reaction forces. The resulting muscle and joint reaction forces were exported and used as loading and boundary conditions for the finite element simulation. Figure 4 shows the DAS multibody model with forearm, wrist, and elbow muscles.

### Finite element model validation

The PMMA material and the PMMA–K-wire contact parameters employed in the present finite element simulations were previously calibrated and validated against four-point bending test reported by Putzer et al. (Putzer et al. 2024). The validation process output was obtained in terms of contact conditions between PMMA and steel K-wires. The validation process is explained in detail in Online Resource, Sect. 1. All simulations were conducted in Ansys Workbench 2022 (Ansys Inc. 2024). The present study focuses on applying the validated parameters to a clinically relevant forearm reconstruction scenario under physiological loading conditions.

### Geometrical reconstruction of the forearm

Three-dimensional geometries of radius and ulna were obtained from medical image segmentation. The patient-specific bone geometry was reconstructed from CT images scan (Siemens Somatom Drive, Siemens Healthineers, Spiral Acquisition, 140 kV, 78 mA, Slice Thickness 1 mm, Exposure Time 1s, Reconstruction diameter 250 mm) using 3D Slicer (version 5.8.1) (Kikinis et al. 2014). Cortical bone was segmented using an intensity-based threshold ranging from 514 to 2108 HU, followed by manual inspection and refinement where necessary. Smoothness factor was set to 0.7. Only the cortical bone was retained for further modeling, while the trabecular bone section was not explicitly represented. This simplification is frequently used in finite element models (Donaldson et al. 2011, Koivumäki et al. 2012).

**Fig. 5:**
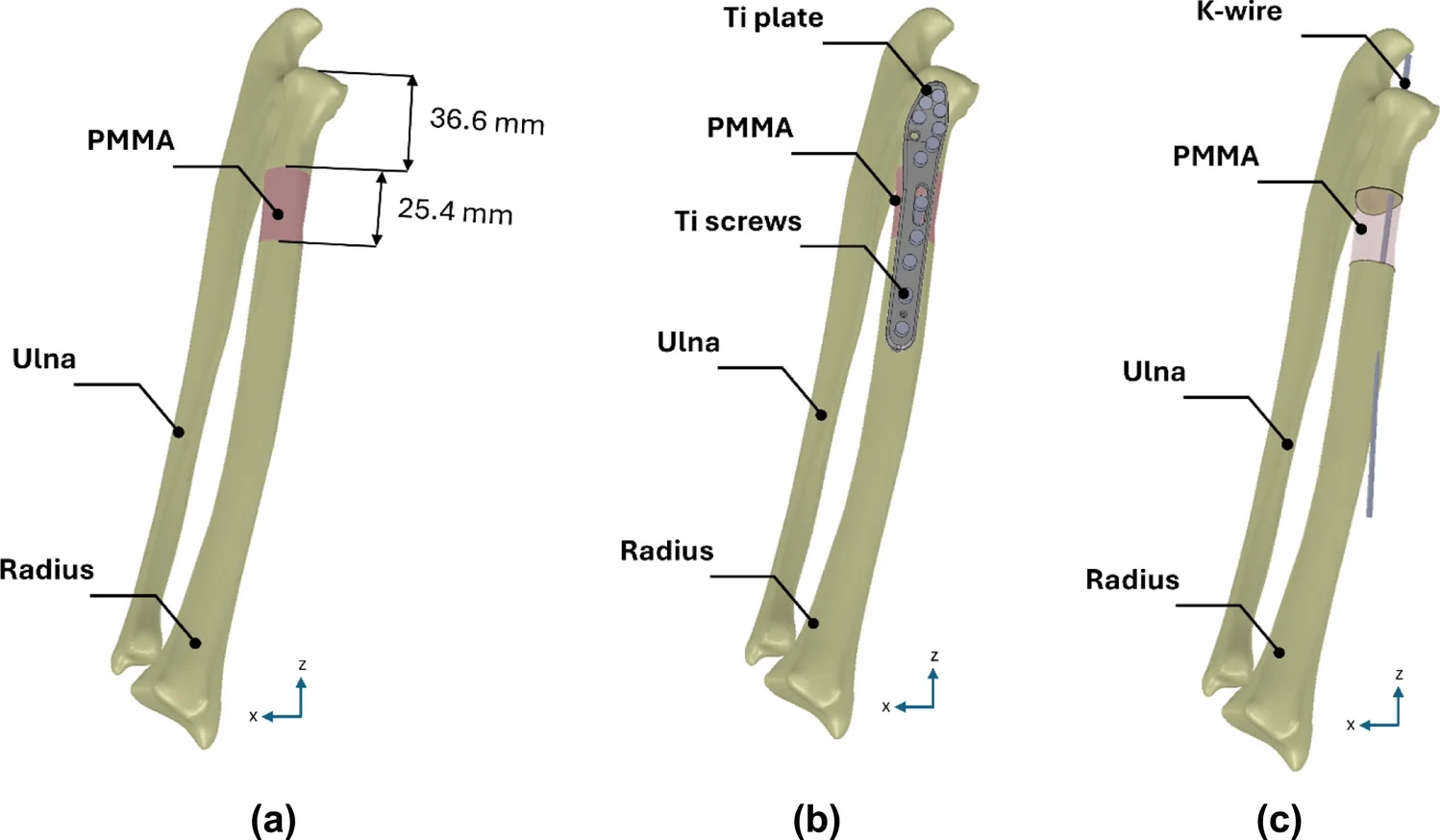
Radius, ulna, and PMMA reconstruction: **a** radius, ulna, and only PMMA with its dimensions; **b** radius, ulna, PMMA, and titanium plate reconstruction; **c** radius, ulna, and K-wire reconstruction

A segment of radius defect was introduced to represent a clinically relevant tumor resection scenario. The defect was reconstructed using PMMA with dimensions shown in Fig. 5a.

Three reconstruction configurations were analyzed:

1. PMMA-only reconstruction (Fig. 5a).
2. PMMA with titanium plate and screws (Fig. 5b).
3. PMMA reinforced with steel K-wire (Fig. 5c).

In the clinical case (Fig. 5b), the titanium alloy APTUS Ankle 2.8/3.5 Distal Fibula Plate (Medartis AG, 2026) was used along with the titanium alloy screws:

- 7 proximal screws with 2.8 mm diameter and length in range 16–20 mm.
- 1 central screw with 2.8 mm diameter and 16.0 mm length.
- 4 distal screws with 3.5 mm diameter and 18.0 mm length.

The screws head and shaft were modeled as simple cylinders. The K-wire (Fig. 5b) of 2.0 mm diameter was positioned to partially penetrate the cortical bone of the radius, providing stability, while the central portion was embedded within the PMMA zone. The K-wire was placed in the tension region of the PMMA block.

### Material properties

Ulna and radius were modeled as linear elastic isotropic cortical bone, with an elastic modulus of 18 000 MPa and a Poisson ratio of 0.30 (Bittner-Frank et al. 2024; Morgan et al. 2018, Rho et al. 1993, Ebacher et al. 2007, Bosisio et al. 2007). PMMA was modeled as linear elastic, with an elastic modulus of 2962 MPa and a Poisson ratio of 0.30, according to experimental results (Putzer et al. 2024, Kühn, 2000, International Organization for Standardization, 2022). The steel K-wire is modeled as stainless steel SS316 with elastic–plastic properties. The elastic region is represented with an elastic modulus of 210 000 MPa and a Poisson ratio of 0.3. Plasticity is represented with an isotropic bilinear hardening model, with a plastic tangent modulus *E*_*t*_ = 1450 MPa, and yield point *σ*_*y*_ = 250 MPa (ANSYS Inc. 2024, Chaouch et al. 2012, Zoric et al. 2022).

The titanium alloy APTUS Ankle 2.8/3.5 Distal Fibula Plate was implemented as linear elastic, with an elastic modulus of 111 200 MPa and a Poisson ratio of 0.34 (Medartis AG, 2026, Bartolomeu et al. 2022, Basir et al. 2023). The screws are represented in the same material as the plate. The material properties are summarized in Table 1.

**Table 1:** Material properties for FEA

Part	Type	Elastic modulus	Poisson ratio	Yield strength	Ultimate tensile strength
Cortical bone	Linear elastic	18 000 MPa	0.30	61–82 MPa	90–130 MPa
PMMA	Linear elastic	2962 MPa	0.30	–	> 50 MPa
Steel K-wire	Bilinear plastic	210 000 MPa	0.30	250 MPa	> 500 MPa
TriLock Distal Fibula plate	Linear elastic	111 200 MPa	0.34	860 MPa	895–930 MPa
Screws	Linear elastic	111 200 MPa	0.34	860 MPa	895–930 MPa

### Mesh properties

**Fig. 6:**
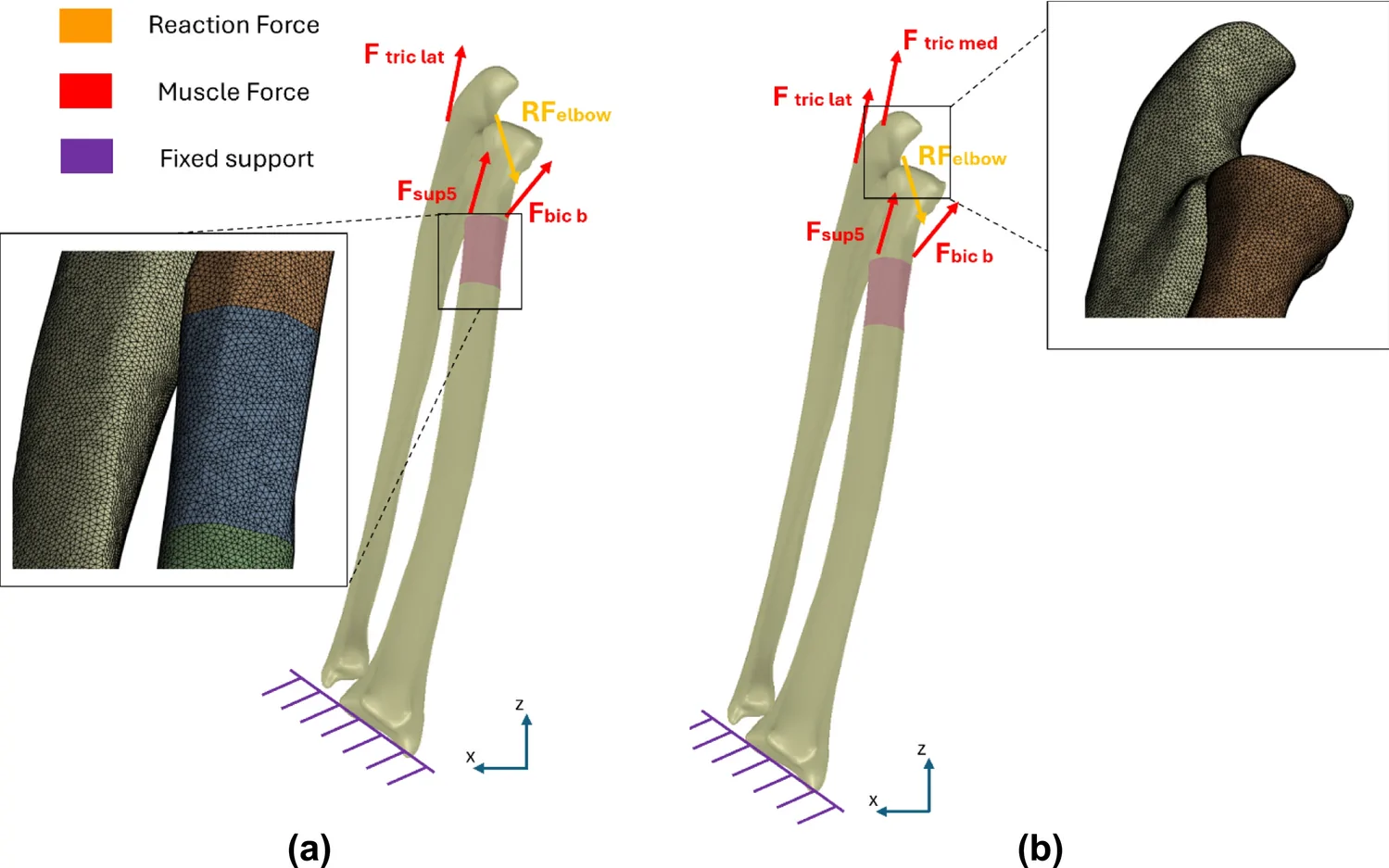
Loads and boundary conditions with the mesh details: **a** case 1–no-weight scenario; **b** case 2–2 kg holding case

All bodies were discretized using second-order tetrahedral solid elements (Ansys SOLID 187), which are well suited for complex anatomical geometries. Mesh density was defined based on the mesh convergence test, monitoring peak stress values. The mesh size value corresponding to variation of less than 5% in the maximum peak von Mises stress value in PMMA and was adopted for all subsequent cases. The three FE models showed comparable mesh densities, ranging from 1.70 to 1.86 million nodes and 1.13 to 1.23 million elements. The mesh details may be seen in Fig. 6.

### Boundary conditions and loading in FE model

Distal surfaces of ulna and radius were constrained to represent physiological fixation, with all degrees of freedom (DOFs) fixed. Muscle forces and elbow joint reaction forces obtained from the multibody musculoskeletal model were applied as loads distributed on localized regions of nodes (circular attachment areas with approximately 5 mm radius) to represent anatomical attachment zones (Fig. 6). The applied loads consisted of biceps brachii, triceps brachii (medial and lateral), and supinator muscle forces, together with elbow joint reaction force. All the muscle forces were applied along the muscle path, defined by origin point (proximal) and distal point (insertion) obtained from the multibody model.

Contact nonlinearity was enabled, and large deformation kinematics were included. All reconstruction configurations were analyzed under identical loading and boundary conditions to ensure compatibility between simulations.

### Contact implementation

Contact parameters were implemented to represent clinical intent, avoiding overconstraining. The bone–PMMA interface was modeled as bonded in the region of distal and proximal cortical contact with PMMA. The K-wire and PMMA interface was modeled as frictional, with a frictional coefficient of 0.95, derived from a validated finite element framework. No direct contact was defined between K-wire and bone outside the penetration region. The screw–plate interfaces were modeled as bonded contacts to represent locking fixation. The screw shafts were bonded to the surrounding bone, assuming ideal fixation without loosening.

To represent the anatomical constraint between radius and ulna, a kinematic coupling was introduced between selected proximal regions of the two bones. A reference node was defined and coupled to nodes of proximal radius and ulna. This approach provides a simplified representation of the stabilizing effect of the proximal radioulnar articulation, which limits relative motion between two bones during functional loading.

## Results

### Musculoskeletal model results

**Fig. 7:**
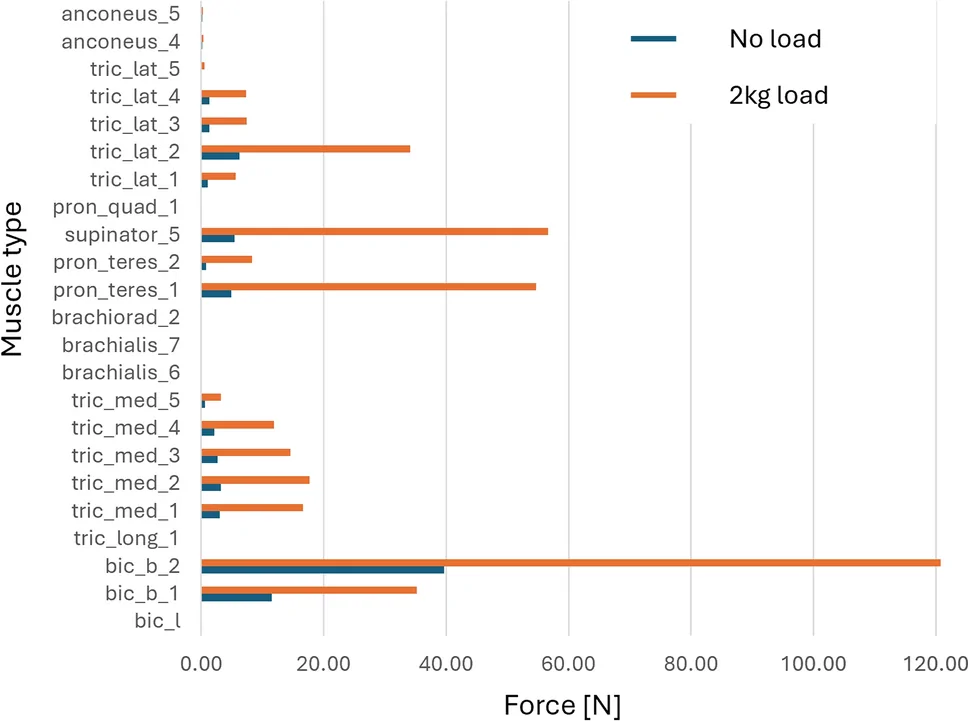
Musculoskeletal model results in terms of muscle forces under no weight applied (blue line), and -19.6 N weight (orange line) applied at the hand (2 kg). Muscle forces corresponding to values below the 5 N threshold were not considered in the simulations

Muscle and joint reaction forces were computed using the DAS model with static optimization and joint reaction analysis for a static holding task with a shoulder flexion of 90° in the sagittal plane. In two loading conditions analyzed in this study, the main muscle contributors to the task were the elbow flexor–extensor muscle group. Figure 7 represents muscle forces for no external load case and for the second holding case.

In the no-load scenario, the predicted muscle distribution showed the biceps brachii that generated the highest force, reaching 39.7 N. Supinator and triceps lateral are in the range of [5–6] N. Most other muscles produced forces below 5 N threshold, with small activation coefficient. The elbow joint reaction force was 71.2 N.

In the holding weight scenario, overall muscle forces increased substantially. The biceps brachii remained the dominant contributor, reaching 120.8 N. Additional muscles that exceed the 5 N threshold are triceps medial with the peak of 17.8 N, triceps lateral reaching 34.2 N, forearm muscle as pronator teres, reaching a peak of 54.7 N, and supinator muscle, with a peak of 56.7 N. The increased magnitude in muscle force confirms the expected rise in demand required to maintain the holding posture under external loading. The elbow joint reaction force was 314.4 N. A complete numerical breakdown of the force values is provided in Online Resource, Sect. 2, Table S1.

### Finite element model results

#### No-weight scenario

**Fig. 8:**
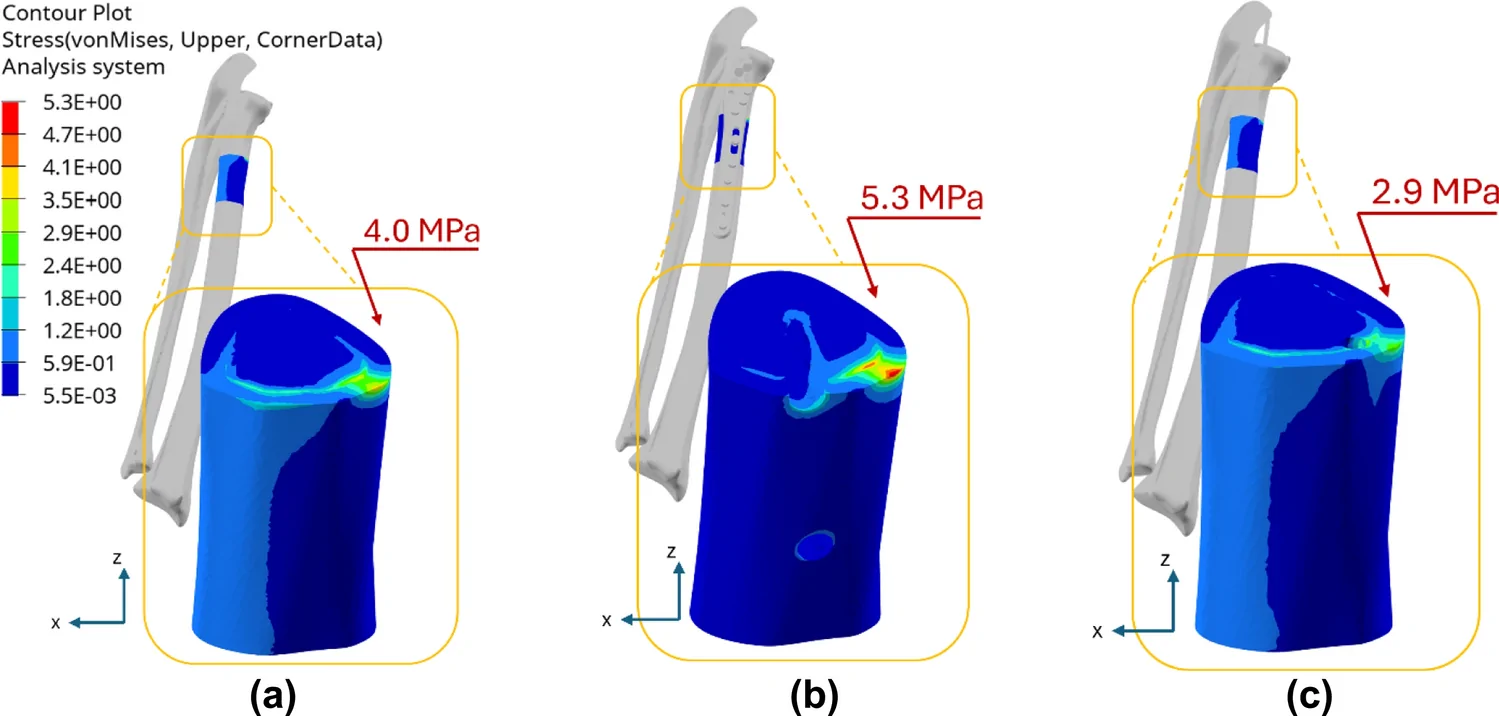
No-load scenario von Mises stress results: **a** only PMMA; **b** PMMA and titanium plate and screws; **c** PMMA reinforced with K-wire

Figure 8 compares the von Mises stress distribution results of the no-weight scenario for three reconstruction study cases: only PMMA, PMMA with titanium plate fixation, and PMMA reinforced with steel K-wire. In all configurations, the highest stress results were concentrated at the medio-proximal transition region, at the PMMA–bone interface. The PMMA-only case exhibited a peak von Mises stress of 4.0 MPa. When the titanium plate–screw construct was introduced, the peak stress in the PMMA increased to 5.3 MPa. In contrast, the reinforced PMMA with K-wire case reduced the peak stress to 2.9 MPa, corresponding to the lowest stress magnitude among the three cases.

Overall, the K-wire reinforcement decreased the peak of von Mises stress by approximately 28% with respect to only PMMA case, and 45% with respect to PMMA with titanium plate fixation.

**Fig. 9:**
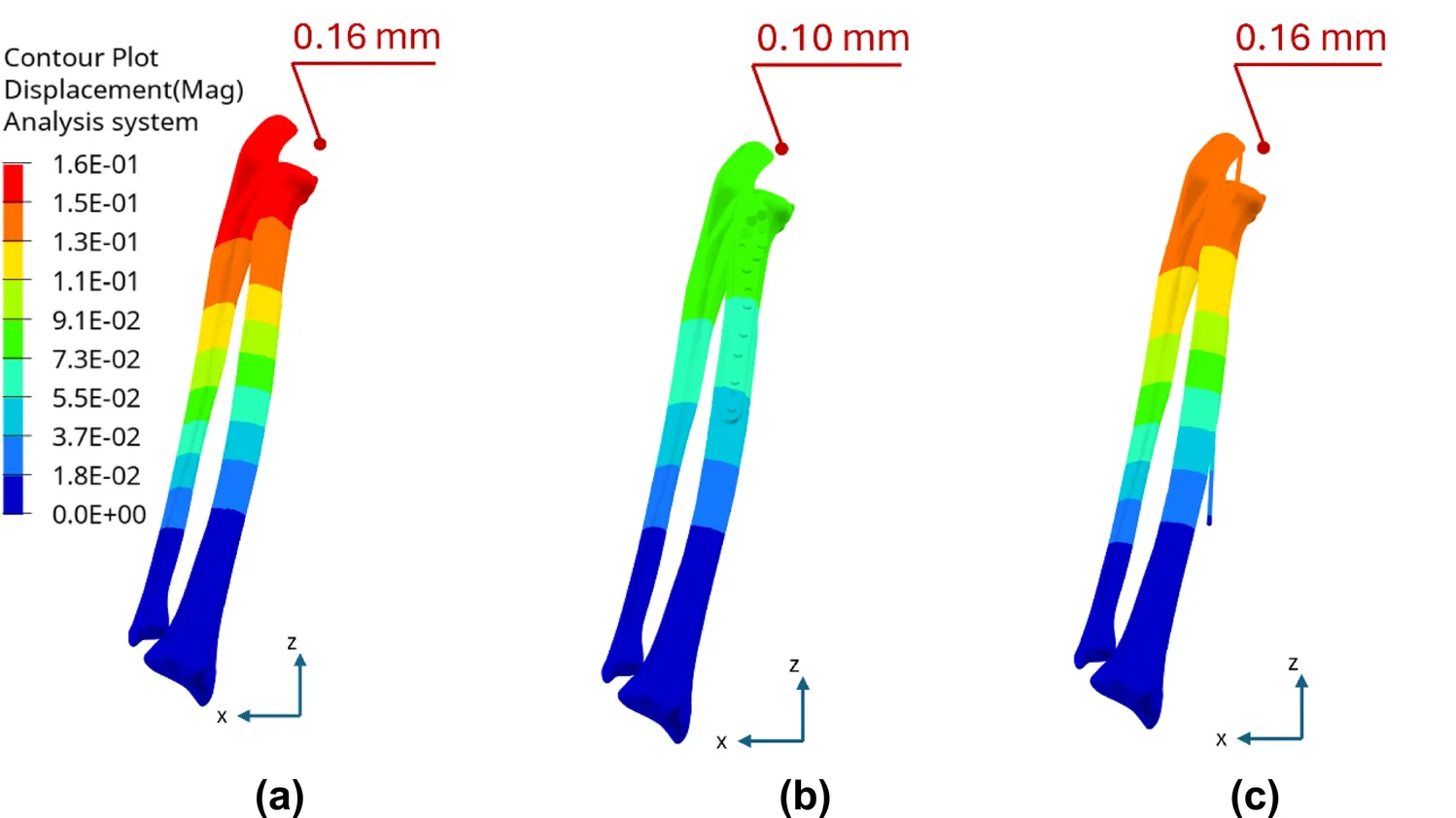
Displacement magnitude for no-weight scenario: **a** only PMMA; **b** PMMA and titanium plate; **c** PMMA reinforced with K-wire

Figure 9 shows the displacement magnitude distribution results of the reconstructed ulna and radius case for three studied fixation methods under the no-weight condition.

Across all models, the maximum displacement occurs at the proximal portion of the forearm, where the elbow joint reaction force is applied. The PMMA-only and K-wire reconstructions exhibited the highest displacement with a peak value of 0.16 mm, indicating the lowest global stiffness of the construct. On the contrary, the titanium plate fixation reduced the peak to 0.10 mm, corresponding to a reduction of 37% compared to PMMA-only case. Overall, the plate fixation method provided the greatest displacement reduction.

#### 19.6 N load scenario

**Fig. 10:**
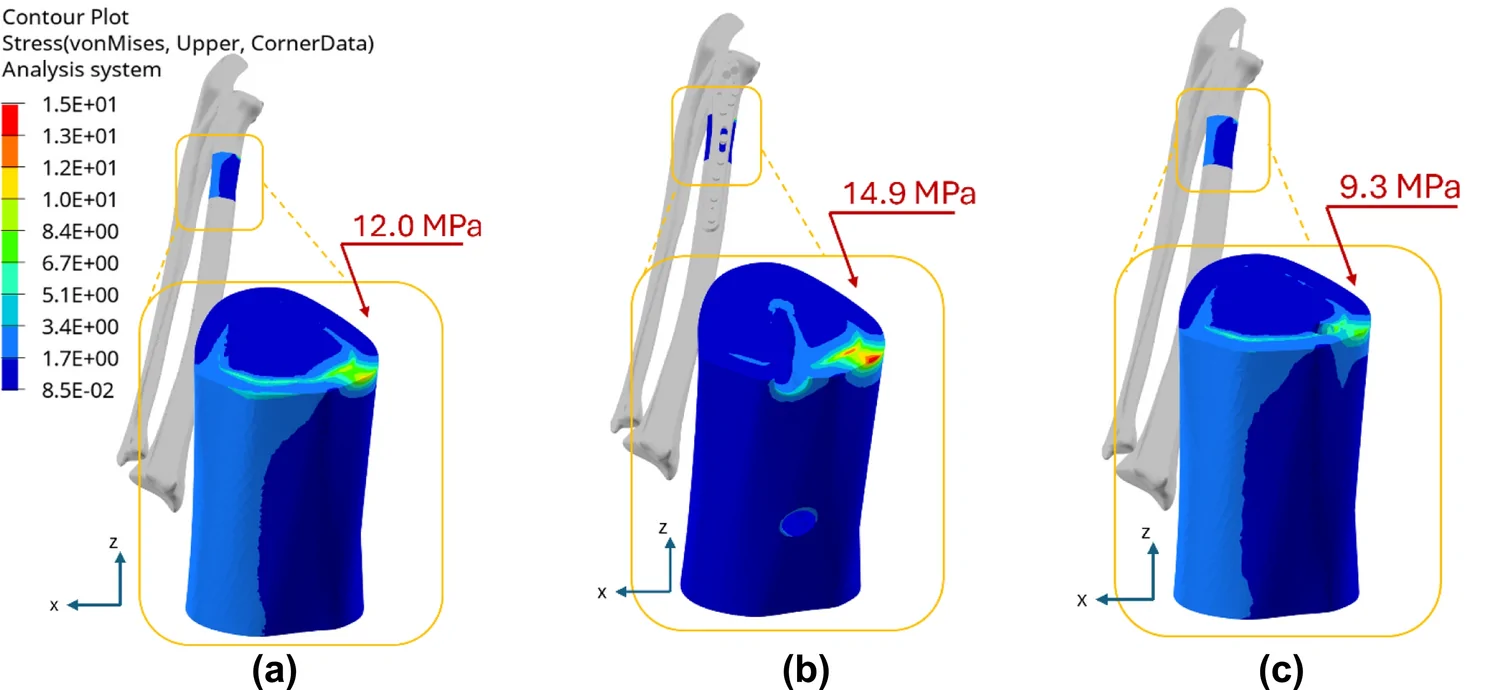
19.6 N loading case von Mises stress results: **a** only PMMA; **b** PMMA and Titanium plate; **c** PMMA reinforced with K-wire

Figure 10 reports the von Mises stress distribution for three PMMA reconstruction cases.

Compared to the no-weight case scenario, the peak stress increased notably on all three reconstruction cases. However, the same trend is observed: Stress concentrations remained localized at the proximal corner of the PMMA–bone interface. The PMMA-only case produced a peak von Mises stress of 12.0 MPa. The titanium plate fixation case increased the peak stress to 14.9 MPa, while the reinforced PMMA with steel K-wire presented a maximum von Mises stress of 9.3 MPa, representing the lowest value among three cases. Overall, the K-wire reinforced reconstruction demonstrated the stress reduction of approximately 23% with respect to PMMA-only case, and 38% compared to the titanium plate fixation.

**Fig. 11:**
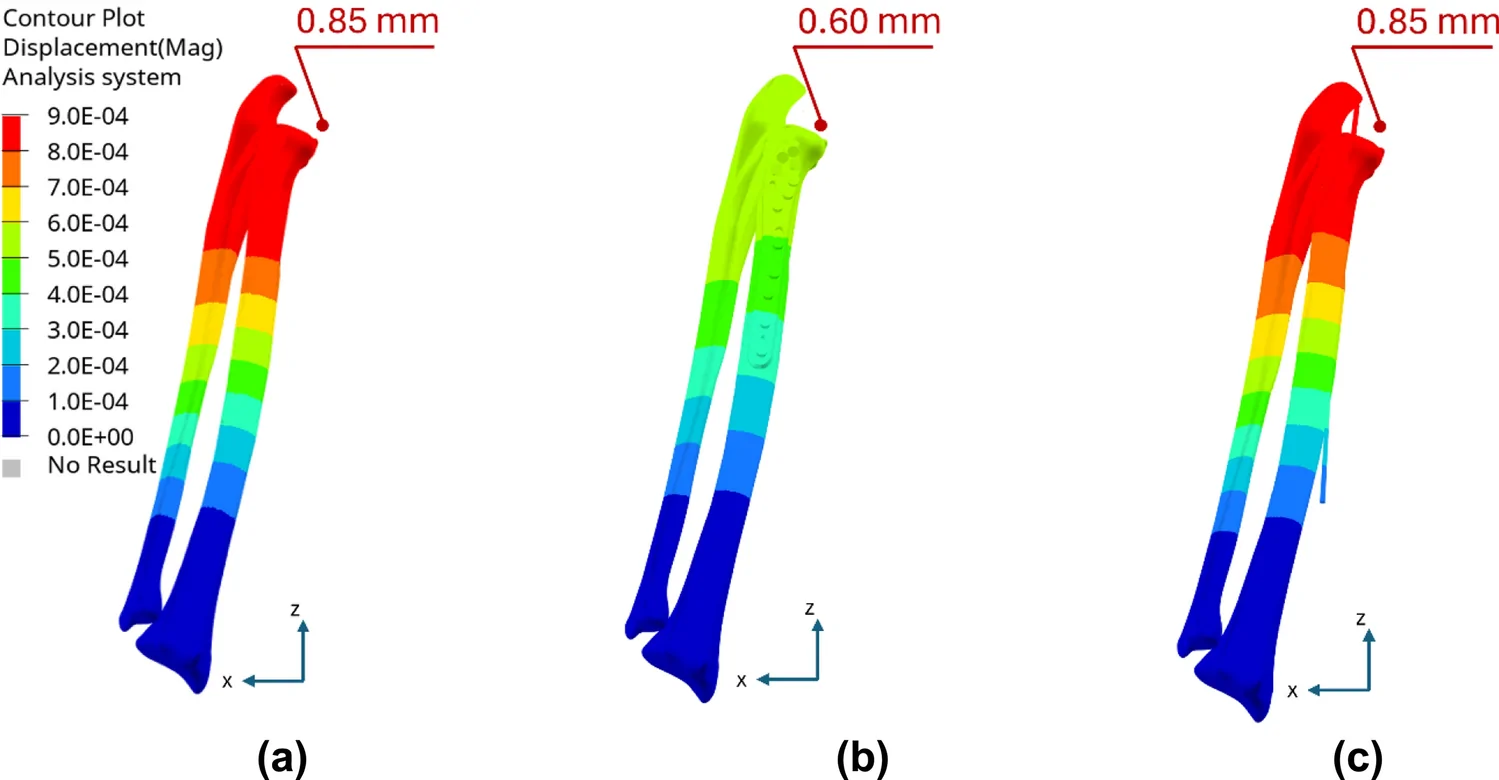
Displacement magnitude for 2-kg loading scenario: **a** only PMMA; **b** PMMA and titanium plate; **c** PMMA reinforced with K-wire

Figure 11 shows the displacement magnitude distribution results of the reconstructed ulna and radius case for three studied fixation methods applying the 2 kg mass at hand.

As in the no-weight scenario, the same pattern may be observed here. The PMMA-only case results in a displacement magnitude of 0.85 mm. Titanium plate-and-screw fixation resulted in the lowest displacement magnitude, reducing the peak to 0.60 mm, corresponding to the reduction of 29% with respect to the PMMA-only case. The reinforced PMMA with K-wire produced 0.85 mm in displacement magnitude, same as PMMA-only. Overall, under functional loading conditions, plate fixation provided the greatest improvement in construct stiffness. Meanwhile, the K-wire case achieved the largest reduction in von Mises stress peak within PMMA.

## Discussion

This study combined musculoskeletal multibody simulation and finite element analysis to evaluate different reconstruction strategies for a clinically relevant proximal radius defect, enabling a comparison of PMMA-only reconstruction, plate fixation, and steel K-wire reinforcement. From a clinical perspective, the present findings support the increasing trend toward augmented cementoplasty and reinforced osteoplasty approaches for oncologic long-bone stabilization. Macro-augmentation strategies to reinforce PMMA have been used in different studies shown in Table 2, where a fixation device was embedded in the cement to buffer the forces PMMA cannot withstand alone. This is particularly important when PMMA is used to fill up bony voids. Although cementoplasty is discouraged in long bones and preclinical tests are substantially lacking, a wide range of reinforcement techniques have been implemented to improve long-bone consolidation via reinforced/augmented osteoplasty in a clinical setting (Table 2).

**Table 2:** Published studies reporting on different consolidative techniques in long bones in a chronological order

First author	Journal	Year	Number of patients	Target bone	Type of intervention	Secondary fractures (%)
Kim et al. (2011)	Surg Oncol	2011	15	Humerus	Ender nail fixation and osteoplasty	NR
Cazzato et al. (2014)	Eur Radiol	2014	51	Long bones	Osteoplasty	9.1
Tian et al. (2016)	CVIR	2014	40	Proximal femur	Osteoplasty (19 patients)	23.8
					Osteoplasty + internal fixation with bone trocars stylets (21 patients)	0
He et al. (2014)	JVIR	2014	6	Proximal femur	Osteoplasty + internal fixation (bone trocars stylets)	0
Kim et al. (2014)	Surg Oncol	2014	15	Femur and tibia	Flexible nailing and osteoplasty	NR
Lin et al. (2015)	Surg Oncol	2015	12	Proximal femur	Osteosynthesis with modified hollow-perforated screws and osteoplasty	8.3
Kelekis et al. (2016)	CVIR	2016	12	Long bones	25–50 stainless steel micro-needles + osteoplasty	0
Liu et al. (2017)	Eur Radiol	2016	36	Long bones	Osteoplasty (19 patients)	26.3
					Osteoplasty + cement-filled catheter in the medullary canal (17 patients)	0
Premat et al. (2017)	Eur Radiol	2017	18	Proximal femur	Spindles + osteoplasty	0
Cazzato et al. (2017)	Eur J Radiol	2017	11	Proximal femur	Osteosynthesis	0
Cornelis et al. (2017)	J Orthop Surg Res	2017	10	Proximal femur	Y-STRUT® device	10
Park et al. (2019)	Skeletal Radiologie	2019	10	Long bones	Multi-hole injection nail (MIN)	0
Lee et al. (2020)	Interv Radiol	2020	6	Proximal femur (4) and tibia (2)	Minimally invasive image-guided ablation, osteoplasty, reinforcement, and internal fixation (AORIF)	NR

Combining two established orthopedic fixation techniques seems like a logical step in fracture control, but only limited knowledge exists on the behavior of the material properties of reinforced bone cement (Putzer et al. 2024). Some of the studies mentioned above and additional case reports suggest safety and non-inferiority of metal-augmented cementing techniques, but their overall effectiveness is yet unclear. In 2020, Kitridis et al. (Kitridis et al. 2020) reviewed 12 studies with almost 350 patients undergoing either metal-augmented or non-augmented percutaneous osteoplasty and found no significant difference between both methods. Augmentation by various metal reinforcements did not seem to alter patient outcome in terms of pain relief, fracture incidence, and operative time when compared to cementoplasty alone. Both techniques appeared equally effective concerning resolution of symptoms, prevention of pathologic fractures, and early facilitation of weight bearing. In patients undergoing augmented cementoplasty, 4% experienced major systemic complications, which were treated successfully but nevertheless accounted for a higher interoperative risk in the reinforced bone cement group. Patients with bone metastases in need of prophylactic surgical fixation are often frail; therefore, the best stabilization technique by the least burdensome approach should be chosen.

The musculoskeletal results demonstrated an increase in both muscle forces and elbow joint reaction force when 2-kg corresponding load was applied. Even modest external load in a holding posture can significantly amplify the mechanical demand on the forearm and elbow muscles. The FE model showed an increase in peak von Mises stress within PMMA from 4.3 to 12.0 MPa in the PMMA-only configuration, highlighting the sensitivity of BC constructs to stress amplification under physiological loads. The K-wire reinforced PMMA construct exhibited the lowest cement stress in both loading scenarios (up to 9.3 MPa in loading case), corresponding to approximately 23% stress reduction with respect to PMMA-only case. The enhanced performance of K-wire reinforcement is mechanically consistent with bending theory, as reinforcement positioned eccentrically toward the tensile side of the PMMA block increases the section resistance to tensile cracking. The peak stress was consistently localized at the proximal region of bone–PMMA interface. This achievement supports the concept that embedding a metallic reinforcement in PMMA, especially when positioned in a tension zone, may reduce stress peak value, enhancing the mechanical properties of BC.

Regulatory standards for acrylic bone cement ISO 5833:2002 (International Organization for Standardization, 2022) specify minimum bending strength of 50 MPa for BC used surgically. In that context, the peak stress observed here remains below typical monotonic bending strength requirements. However, cement failure in vivo is frequently governed by fatigue and crack propagation, where allowable stress amplitude is substantially lower than allowed bending strength. Several studies on fatigue life of acrylic bone cement shows the infinite life (> 1 000 000 cycles) for stress levels in the range 10–20 MPa in uniaxial tensile tests (Lewis and Li 2010). However, fatigue life of BC highly depends on stress magnitude and specimen variables. Moreover, mechanical properties of PMMA decrease furtherly when defects and porosity are observed.

Plate fixation provided the greatest reduction in global displacement (0.85 mm only PMMA case vs. 0.60 mm with plate under load), indicating superior construct stiffness and stabilization, but showed higher peak stress in PMMA. These findings highlight a clinically relevant trade off: Plate fixation most effectively limits overall deformations, whereas internal reinforcement most effectively reduces peak stress within PMMA, which may be advantageous for limiting cement cracking risks.

However, several limitations should be considered when interpreting these results. This work presents a single case patient-specific study, so the reinforced PMMA performance depends on defect geometry, cement volume, cortical thickness, and implant positioning. Next, several interfaces were modeled as bonded: bone–PMMA regions, screws–bone, and screw–plate. The use of bonded contact represents an idealized fixation scenario and may overestimate the degree of constraint, potentially leading to higher local stress prediction, and may underestimate progressive loosening effect that could occur clinically. Then, the present evaluation was focused on quasi-static loading; further studies may explore fatigue loading, fracture or damage model. The present study should be interpreted as a patient-specific computational framework for the biomechanical assessment and preoperative planning of tumoral fracture cases requiring cementoplasty. Within this framework, different augmentation strategies were evaluated under identical boundary conditions, including bone cement application alone and in combination with plate fixation or intra-PMMA K-wire reinforcement. This approach enables a controlled comparison of their relative mechanical performance in terms of stress distributions within the same anatomical and loading environment. Moreover, the objective is not to derive generalized clinical recommendations, but rather to support patient-specific decision-making by quantifying the biomechanical consequences of alternative fixation configurations. In this context, the proposed K-wire reinforced bone cement configuration is introduced as an exploratory augmentation strategy that may lead to further investigation in more extensive parametric, multi-patient, and eventually experimental studies.

## Conclusions

In this study, the performances of reinforced bone cement with steel K-wire were evaluated in clinically relevant configurations. Reinforcing cement with an embedded K-wire produced the lowest peak in PMMA stress distribution in both loading scenarios, compared to PMMA-only case under physiological loading. Plate fixation provided the greatest construct stiffness reducing the maximum displacement. Overall, these findings indicate that K-wire reinforcement and plate fixation offer complementary mechanical benefits. Future work should extend this framework to include fatigue analysis and wire placement sensitivity to further optimize reinforcement strategies and improve long-term reconstruction reliability.

## Supplementary Information

Below is the link to the electronic supplementary material.

Supplementary file1 (DOCX 197 KB)

## Data Availability

No datasets were generated or analysed during the current study.

## Declarations

### Generative artificial intelligence

Artificial intelligence tools (ChatGPT 5.2) were used to support language editing and improve clarity and readability of the manuscript. No AI tools were used in study design, data collection, data analysis, data interpretation, or figure generation. All scientific content, analyses, and conclusions are the sole responsibility of the authors.
