# Supplementary material for: An analysis of different reconstruction techniques for the radius: a finite element analysis
Source: Biomech Model Mechanobiol. 2026 Sep 28;25(5):115. doi: 10.1007/s10237-026-02116-z (PMC13619793; doi:10.1007/s10237-026-02116-z)
Supplement: Supplementary file 1 [file 10237_2026_2116_MOESM1_ESM.docx]

## Supplementary Information

**Section 1 Validation of finite element model through experimental four-point bending test**

A finite element model of reinforced PMMA was validated against experimental data of four-point bending test from previous work (Putzer, 2024). The authors investigated different reinforcement strategies of PMMA: K-wire and steel cable with pretensioning. They conducted a four-point bending test on at least 5 specimens per category. The servo-hydraulic material testing machine was used with a constant cross-head speed of 5mm/min. The specimens were tested until failure, determined by a load drop exceeding 50 N. Reinforcement K-wire presented a diameter of 2.0 mm. The volume of the reinforcement to total volume of the sample was 2.1 %. The overall dimensions of the specimens were 15x10x200 mm, with tolerance ±1.0 mm. The reinforcement was placed in tension zone, at 2.4 mm from the lower surface of PMMA specimen.

A finite element model of four-point bending simplified setup was develop to replicate experimental test configuration. A PMMA block geometry reinforced with K-wire was developed in SolidWorks 2022.1. The support and loading rollers were positioned at the same span as in experimental testing, and the load was applied with two loading rollers positioned symmetrically in respect to the midspan of the specimen. Figure S1 represents simulated four-point bending setup.

**Fig. S1** Simulated four-point bending setup

The PMMA material properties were obtained from experimental results. In finite element model, the PMMA block was modeled as linear elastic, with E = 2962 MPa, and Poisson ratio of 0.3. The steel K-wire was modeled with elastic and plastic behavior to represent the experimentally observed non linear response. Elasticity modulus of steel component is 210 000 MPa, and Poisson ratio of 0.3. The plasticity was implemented as isotropic bilinear hardening model, with yield stress of 250 MPa and tangent modulus E_t_ = 1450 MPa.

A key validation goal was to determine the contact conditions between PMMA and steel reinforcement. Therefore, different interaction definitions between PMMA and K-wire were evaluated:

- bonded contact: perfect adhesion, no slip or separation allowed
- frictionless contact: free sliding
- frictional contact with coefficient ranging in [0.1 - 1.5]

The influence of each contact assumption was evaluated in terms of peak von Mises stress on cable and PMMA block and confronted against experimental results.

The frictional model provided the closest agreement between experimental and simulated data. The friction coefficient of 0.95 was chosen (Figure S2).

**Fig. S2** Experimental vs numerical results of four-point bending setup

The selected frictional coefficient of 0.95 reproduces an effective interaction between PMMA and steel K-wire under bending. Although local mechanical microslips may occur, the calibrated value enabled the numerical model to reproduce effectively the experimentally observed macroscopic behavior.

**Section 2 Validation of finite element model through experimental four-point bending test**

Table S1 summarizes the results in terms of muscle forces obtained from the multibody musculoskeletal model that were subsequently applied in finite element analysis.

**Table S1** Muscle and joint reaction forces

| **Muscle forces and joint reaction forces** | **Force [N]** | |
| --- | --- | --- |
|  | **No-weight scenario** | **2 kg holding scenario** |
| Bic_b_1 | 11.6 | 35.2 |
| Bic_b_2 | 39.7 | 120.8 |
| Tric_med_1 | / | 16.6 |
| Tric_med_2 | / | 17.8 |
| Tric_med_3 | / | 14.6 |
| Tric_med_4 | / | 11.9 |
| Pron_teres_1 | / | 54.7 |
| Pron_teres_2 | / | 8.3 |
| Supinator_5 | 5.4 | 56.7 |
| Tric_lat_1 | / | 5.6 |
| Tric_lat_2 | 6.2 | 34.2 |
| Tric_lat_3 | / | 7.5 |
| Tric_lat_4 | / | 7.3 |
| Joint reaction force | 71.2 | 314.4 |
